# Epirubicin Enhances the Anti-Cancer Effects of Radioactive ^125^I Seeds in Hepatocellular Carcinoma *via* Downregulation of the *JAK/STAT1* Pathway

**DOI:** 10.3389/fonc.2022.854023

**Published:** 2022-05-27

**Authors:** Lei Guo, Jiali Sun, Changjun Wang, Yang Wang, Ya Wang, Dong Li, Yuliang Li

**Affiliations:** ^1^ Department of Vascular Anomalies and Interventional Radiology, Qilu Children’s Hospital, Cheeloo College of Medicine, Shandong University, Jinan, China; ^2^ Department of Vascular Anomalies and Interventional Radiology, Jinan Children’s Hospital, Jinan, China; ^3^ Shandong Provincial Clinical Research Center for Children’s Health and Disease, Jinan, China; ^4^ Department of Interventional Radiology, Jiyang People’s Hospital of Jinan, Jinan, China; ^5^ Department of Interventional Medicine, The Second Hospital, Cheello College of Medicine, Shandong University, Jinan, China; ^6^ Institute of Interventional Oncology, Shandong University, Jinan, China

**Keywords:** radioactive ^125^I seeds, Epirubicin, *JAK/STAT1* pathway, hepatocellular carcinoma, anti-cancer effect

## Abstract

The application and promotion of ^125^I seed implantation technology have increased the safety and effectiveness of the clinical treatment of advanced hepatocellular carcinoma (HCC). Epirubicin (EPI) is a traditional anthracycline chemotherapy agent that has minimal side effects and has been widely used in the clinical treatment of HCC. We hypothesized that EPI would enhance the anti-cancer effects of ^125^I seeds *via* the *JAK/STAT1* signaling pathway. Thus, we aimed to investigate whether EPI could enhance the radiosensitivity of HCC cells to ^125^I and determine the underlying molecular mechanism. This basic study was conducted in an animal laboratory at Shandong University. BALB/C male nude mice were used, and all animals were fed and treated according to the standards of the Institutional Animal Care and Use Committee of Shandong University. Both *in vitro* and *in vivo* models of ^125^I irradiation of HCC cells were created. The anti-cancer effects of ^125^I and the role of EPI in promoting these effects were evaluated using flow cytometry for apoptosis and cell cycle, CCK-8 assay for EPI drug cytotoxicity, and transwell assays for migration and invasion. The potential mediating effect of the *JAK/STAT1* pathway was assessed using an isobaric tag for relative and absolute quantitation analysis to identify differentially expressed proteins after ^125^I treatment. Transfection of HCC cells with *STAT1*-RNAi were performed to determine the effect of *STAT1* downregulation on ^125^I and EPI treatment effects. The radiosensitivity concentration of EPI promoted ^125^I-induced anti-cancer effects, including apoptosis, anti-proliferation, and inhibition of migration and invasion. These effects were mediated *via* the *JAK/STAT1* pathway. Downregulation of *STAT1* compromised measured anti-cancer effects, which were both confirmed in the *in vivo* and *in vitro* models. EPI can promote ^125^I-induced anti-cancer effects in HCC. The *JAK/STAT1* pathway may be a potential target for ^125^I seed implantation in the treatment of HCC.

## Introduction

Hepatocellular carcinoma (HCC) is the third most general malignancy of the digestive system and can induce cancer-related death (1, 2). The prognosis of HCC is usually poor, and with the 5-year survival rate being lower than that for prostate, breast, and lung cancers, HCC has a serious negative effect on patients’ quality of life (1). Although the incidence rates of other cancers have decreased over the past decade, the incidence rate of HCC has continued to increase, especially among women, at a rate of 2.1% per year (1, 2). The use and promotion of iodine 125 (^125^I) seed implantation technology have increased the safety and effectiveness of the clinical treatment of advanced HCC, especially when used in combination with other chemotherapy drugs, such as lobaplatin, and traditional Chinese medicine preparations, with significant improvement in prognosis (3–5). Nevertheless, the concrete mechanism underlying the effect of ^125^I seeds on HCC cells remains to be completely described. In addition, it is unclear whether chemotherapy drugs can enhance the radiation sensitivity of HCC to ^125^I seeds. Exploring the mechanism of action and new targets of ^125^I seeds would establish a sound foundation for the preferable clinical efficacy of ^125^I seeds and provide new therapeutic ideas for HCC.

It is well known that any abnormality or alteration of signaling regulators may lead to tumor formation. In this way, signal transducer and activator of transcription 1 (*STAT1*) can play an essential role in signal transduction induced by many cytokines (6). STAT1 is mainly involved in antiviral and antibacterial reactions, inhibits tumor growth, and induces cell apoptosis by regulating anti-apoptotic genes such as *Bcl-XL*, caspases, and *Bax* (7, 8). Research has shown that abnormalities in 12 signaling pathways mainly relate to the occurrence and development of cancer, with the *JAK/STAT1* signaling pathway being one of these pathways (9). The *JAK/STAT1* signaling pathway might regulate the metastasis of solid tumors, with mutations of the *JAK/STAT1* pathway being closely related to tumorigenesis (6).

Epirubicin (EPI) is a traditional anthracycline chemotherapy agent that has minimal side effects and thus has been widely used in the clinical treatment of breast, liver, and gastric, and non-small cell lung cancer (NSCLC) (10, 11). EPI inhibits the proliferation of tumor cells by interfering with the DNA transcription process and inhibiting the synthesis of DNA and messenger RNA (12). In clinical practice, EPI is often used in combination with other drugs or vectors, such as trastuzumab, paclitaxel, polymer micelles, and hyaluronic acid, to enhance anti-tumor effects (12, 13). However, the mechanism of EPI when combined with ^125^I seeds for the treatment of HCC remains unknown. Therefore, identifying the mechanism and function of EPI in the near irradiation of ^125^I seeds is a clinically significant issue.

In this study, we aimed to evaluate whether EPI could enhance the radiosensitivity of HCC to ^125^I and determine the underlying molecular mechanism. The results of the isobaric tag for relative and absolute quantification (iTRAQ) analysis and assessment of the features of the *JAK/STAT1* signaling pathway indicated that ^125^I seed might induce apoptosis of HCC cells by upregulating the *JAK/STAT1* signaling pathway. In addition, we revealed that EPI could enhance ^125^I seed-induced apoptosis and anti-proliferation activity. Based on these findings, we suggest that the EPI-induced enhanced anti-cancer effects of ^125^I seeds might be mediated by the *JAK/STAT1* signaling pathway.

## Materials and Methods

### Statement of Ethics

BALB/C male nude mice were used. All animals were raised and processed according to the standards of the Animal Care and Utilization Committee of Shandong University. The animal study was reviewed and approved by Shandong University, and the approval number is KYLL-2021(LW) 091.

### Nude Mice Xenograft Tumor Model

The SMMC7721 cells were transfected with NC-RNAi (Negative control-RNA interference, TTCTCCGAACGTGTCACGT) or *STAT1*-RNAi (GAGCAGGTTCACCAGCTTTAT) using 0.9% normal saline solution to resuspend fresh sterile cell suspensions (1×106 cells/well) and injecting them into the left hind limb of the mice.

Once the volume of the xenografts reached 400 mm^3^, the mice were randomly allocated to the relevant experimental groups, as described below. There are 25 mice used in this experiment and 5 mice per group. Volume and weight were measured using Vernier calipers and a digital scale, respectively. Tumor volume was calculated as follows: length × width^2^ × 0.5. The tumor was stripped and lysed, and RNA was extracted when the mice were killed.

### Implantation of Radioactive ^125^I Seeds and Radiation of HCC Cells

The skin over the tumor site was sterilized with an iodine disinfectant, anesthetized with lidocaine, and then punctured in the center of the tumor using an 18-G needle (Kakko, Japan). ^125^I seeds (Ningbo Junan Pharmaceutical Technology Company, China) were then implanted into the tumor using a seed implant device. After implantation, a sterile cotton swab was used to apply pressure to stop the bleeding. The *in vitro* radiotherapy model used was based on previous studies, with an initial activity level of 3.0 mCi and a dose rate of 3.412 cGy/h (4).

### Cell Lines and Lentiviral Transfection

The HCC cell lines SMMC7721 and HepG2 were obtained from the Zhong Qiao Xin Zhou Biotechnology (China). SMMC7721 cells were cultured in RPMI 1640 (Corning, Inc., Corning, NY, USA) supplemented with 10% fetal bovine serum (FBS) and 1% penicillin-streptomycin. HepG2 cells were cultured in Dulbecco’s modified Eagle’s medium (Corning, Inc., Corning, NY, USA) supplemented with 10% FBS and 1% penicillin-streptomycin. Cells were cultured at 37°C in 5% CO2. Lentivirus (GeneChem, Shanghai, China) was used for STAT1 knockout. The most appropriate multiplicity of infection was 10, as recommended by the protocol. Complete medium, HiTransG A (GeneChem, Shanghai, China), and lentivirus were mixed and then added to inoculated cells on 6-well plates for transfection. After transfection for 16 h, the medium was replaced with complete culture medium containing 2.5 μg/mL puromycin, and the cells were incubated for 48 h. The concentration of puromycin was then successively reduced to complete the screening. The transfection efficiency was confirmed using western blot after 72 h.

### iTRAQ Labeling

RIPA (TIANGEN, Beijing, China) was used to extract total protein from ^125^I-irradiated SMMC7721 cells and negative control cells. Jiyun Biotech (Shanghai, China) was responsible for iTRAQ labeling. Ingenious pathway analysis (INGENUITY) was used for the signal pathway enrichment and biofunction analysis. A fold change in the mean value of labeling of >1.2 and a P-value <0.05 between the ^125^I-treated and control groups indicated significant upregulation.

### Cell Sensitivity Selection For EPI

The sensitivity of SMMC-7721 and HepG2 cells to EPI drug cytotoxicity was assessed using the cell counting kit-8 (CCK-8; Dojindo, Japan). Cells cultured in 96-well plates were treated with EPI at concentrations of 0–5 μg/mL for 72 h. The cell number of control (N_C_) and EPI (N_EPI_) group were used to calculate the inhibition rate (1-N_EPI_/N_C_). The half-maximal inhibitory concentration (IC50) was calculated by Prism 9.0 (GraphPad, San Diego, USA), and 10% of the IC50 was selected as the EPI-sensitization concentration.

### Cell Proliferation Assay

The treated cells were prepared into a cell suspension at a concentration of 3×10^3^/ml and placed into 96-well plates to achieve a cell suspension volume of 200 µL/well. CCK-8 reagent was added at 0 h, 24 h, 48 h, and 72 h, and the cells were incubated at 37°C for 2 h. The absorbance of each well was scanned by a microplate reader (Thermo, Waltham, MA, USA) at a wavelength of 450 nm.

### Flow Cytometry for Cell Cycle and Apoptosis Analysis

Cells were cultured in 6-well plates (2×10^5^ cells/well) and collected after treatment. For apoptosis analysis, 5 μL Annexin V–APC (Elabscience, Wuhan, China) or Annexin V–FITC (BD Biosciences) and propidium iodide (PI) were added to the cells using binding buffer for staining, with the detection performed after 20 min of darkness. For cell cycle analysis, cells were fixed in pre-cooled ethanol for 1 h. Before analysis, the cells were stained with PI and RNase A (BD Biosciences, Franklin Lakes, NJ, USA). Cell cycle and apoptosis assays were performed using a flow cytometer (Beckman, USA).

### EDU Staining

A Cell-Light EDU Apollo567 *In Vitro* Kit (RiboBio, Guangdong, China) was used to detect cell proliferation. According to the manufacturer’s instructions, reagent A and complete medium were diluted to a 1:1000 concentration and incubated at 37°C for 2 h. Next, the cell nuclei were stained using Hoechst 33342. Finally, a fluorescence microscope (Olympus, Tokyo, Japan) was used to observe and photograph cells, with Image J used to count the number of cells.

### Western Blot and Antibodies

The treated cells were washed twice with cold 1X phosphate-buffered saline, lysed with RIPA lysate buffer for 20 min, and centrifuged (12,000 rpm, 10 min, 4°C) to obtain the supernatant. The loading buffer was added, and the supernatant was denatured at 95°C for 5 min until protein denaturation was achieved. Electrophoresis analysis of protein samples (10 μL/well) was performed using 10–12% sodium dodecyl sulfate-polyacrylamide gel electrophoresis.

Proteins were transferred onto nitrocellulose membranes (Millipore, Burlington, MA, USA). The blots were blocked with 5% nonfat milk powder for 1 h and incubated with corresponding primary antibody at 4°C overnight. On the second day, the blots were cleaned with 1X tris-buffered saline and incubated with the appropriate secondary antibodies at room temperature for 1 h. Protein brands were detected by chemiluminescence (Millipore, Burlington, USA), and the expression level was determined using Image J.

Primary antibodies against *JAK* (ab133666), anti-*p-JAK* (ab138005), anti-*STAT1* (ab109320), anti-*p-STAT1* (ab109461), *mTOR* (ab32028), *p-mTOR* (ab109268), *p-AKT* (ab105731), *Bax* (ab32503), and *Bcl2* (ab182858) were purchased from Abcam (Cambridge, UK). The following secondary antibodies were purchased from GenScript (Piscataway, NJ, USA): goat anti-rabbit IgG (H&L) and goat anti-mouse IgG (H&L).

### Invasion and Migration Assays

Cell invasion and migration were assessed using transwell assays. For the invasion assay, Matrigel (BD Biosciences) and Opti-MEM (Gibco, USA) were mixed at a ratio of 1:5, and 50 μL of the mixture was then placed in a Boyden chamber (BD Biosciences, Bedford, MA) and incubated at 37°C for 1 h. Complete medium containing 30% FBS (600 μL) was added to each well of the 24-well plates. Then, 200 μL cell suspension (2×105) was added into the Boyden chamber and incubated at 37°C for 24 h. Cells were fixed with 4% paraformaldehyde, stained with crystal violet for 30 min, and washed gently with water twice. Cells were photographed using an inverted microscope (Olympus CKX53, Tokyo, Japan) and counted using ImageJ software. The procedure of the migration assay was identical to that of the invasion assay, with the exception of Matrigel.

### Immunohistochemistry

For IHC staining, the samples were incubated in primary antibody against p-STAT1 (1:200, ab109461, Abcam) following standard procedures. All images were acquired by Nanozoomer Digital Pathology Scanner (Hamamatsu, Japan), and integrated optical density (IOD) and area were measured by using Image Pro Plus 6 AMS software. Mean density (IOD/area) was used to evaluate the expression level.

### Statistical Analysis

All results were repeated at least three times. All data are expressed as mean ± SD. Statistical analyses were performed using GraphPad Prism software version 9.0. One-way analysis of variance, with Tukey’s test for multiple comparisons, was used to evaluate the differences between more than two preselected groups, with an independent sample *t*-test used to analyze the statistical significance between the two groups. P-values <0.05*, <0.01**, and <0.001*** were significant. The statistical methods of the study were reviewed by Bin Liu from Shandong University.

## Results

### Enhancement of the ^125^I-Induced Anti-Proliferation Effect of HCC Cells by EPI

**Figure 1:**
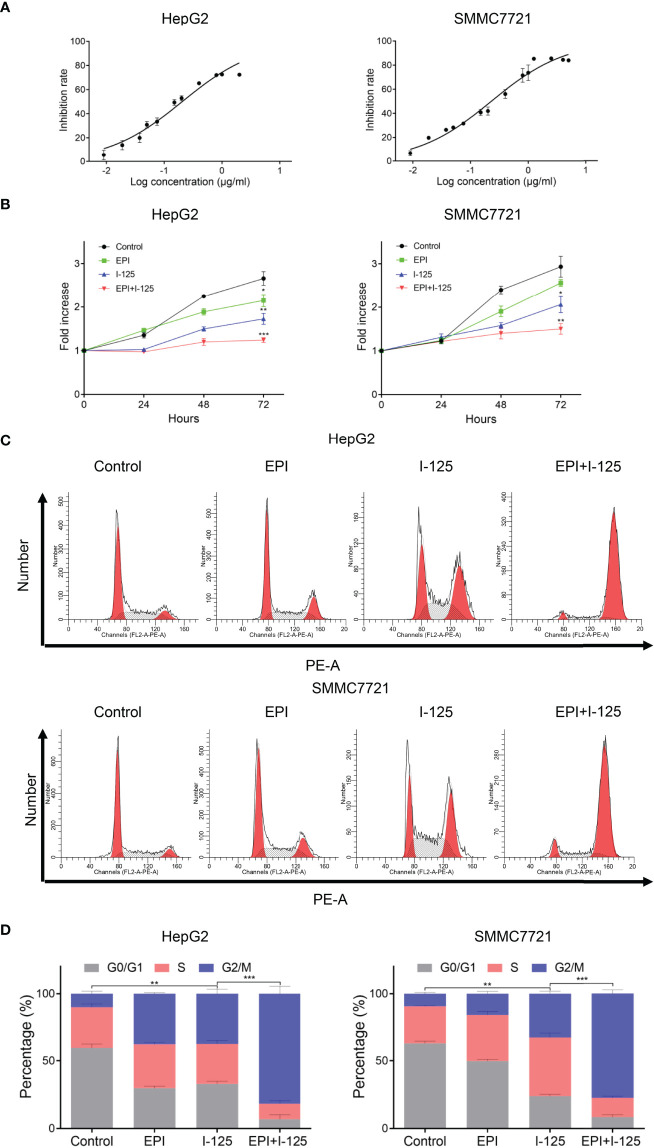
EPI enhances the ^125^I-induced anti-proliferation effect in HCC cells. **(A)** Inhibition rate of EPI on HCC cells. **(B)** CCK-8 assay quantifying HCC cell proliferation after EPI and ^125^I treatment, alone or in combination. **(C, D)** Cell cycle analysis after EPI and ^125^I treatment, alone or in combination. All experiments were performed in triplicate, and the data are presented as the mean ± SD. *P < 0.05, **P < 0.01, ***P < 0.001. HCC, hepatocellular carcinoma cells; EPI, epirubicin; CCK-8, cell counting kit-8.

The effect of EPI in improving the ^125^I-induced anti-proliferation impact in HCC cells, detected using CCK-8 assay in HepG2 and SMMC7721 cell survival curve, is shown in **Figure 1A**. EPI dose-dependently inhibited the proliferation of HepG2 and SMMC7721 cells, with EPI-sensitization concentrations of 0.020 µg/ml and 0.023 µg/ml, respectively. The fold increase rate of HepG2 and SMMC7721 cells was greater with the combined use of EPI and ^125^I seeds than with EPI alone and the difference between ^125^I and combined group was statistically significant (**Figure 1B**). In HepG2 and SMMC7721 cell, the fold increase rate of ^125^I at 72 h was 1.726 ± 0.126 and 1.980 ± 0.284, while combined group was 1.240 ± 0.055 and 1.500 ± 0.122. The effects of EPI and ^125^I, alone or in combination, on the cell cycle are shown in **Figure 1C**. Consistent with the cell proliferation assay, the cell cycle G2/M arrest was greater in the combined test group than in either the single ^125^I or EPI treatment group (**Figure 1D**).

### Enhancement of ^125^I-Induced Cell Apoptosis And Inhibition of Cell Migration and Invasion by EPI

**Figure 2:**
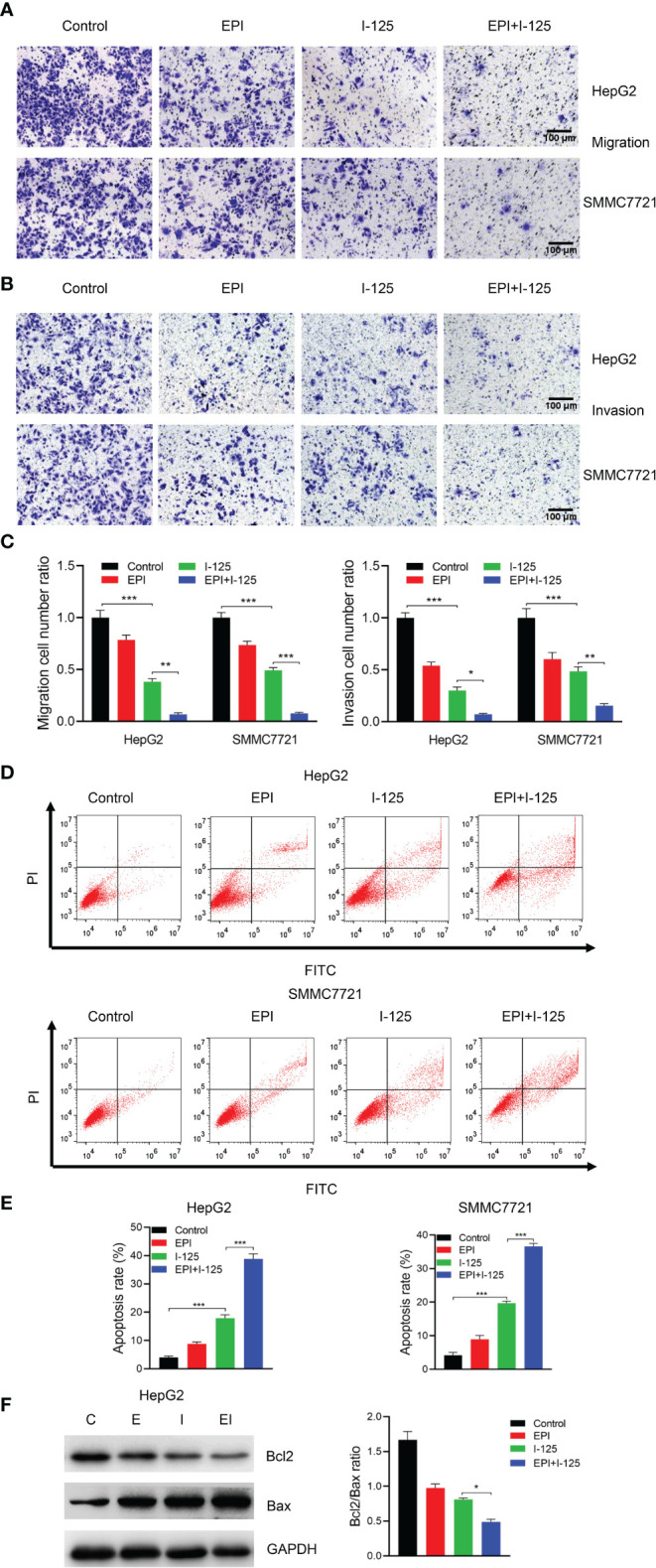
EPI enhances the inhibition effect of ^125^I on cell migration and invasion and induction of cell apoptosis. **(A–C)** Transwell assays showing the effect of EPI in promoting ^125^I-induced inhibition of migration and invasion of HCC cells. **(D, E)** Flow cytometry showing the combined effect of EPI and ^125^I. **(F)** Western blot showing the Bcl2/Bax ratio. All experiments were performed in triplicate, and the data are presented as the mean ± SD. *P < 0.05, **P < 0.01, ***P < 0.001. HCC, hepatocellular carcinoma cells; EPI, epirubicin; Bcl2, Bcl2 apoptosis regulator; Bax, Bcl2 associated X.

The results of the transwell assays of HCC cells after treatment with EPI and ^125^I, used alone or in combination, performed to quantify the effects of EPI in promoting ^125^I-induced effects on HCC cell migration and invasion, are shown in **Figures 2A–C**. The number of cells in the migration and invasion phases was significantly lower in the combined test group than in either the individual ^125^I or EPI test group. Furthermore, the Annexin V–FITC/PI assay, implemented to evaluate the effect of EPI on ^125^I-induced apoptosis of HCC cells, showed a significantly higher rate of apoptosis in the combined test group than in either the EPI or ^125^I individual treatment groups (**Figures 2D–F**). Overall, EPI promoted the inhibitory impact of ^125^I on cell invasion and migration and promoted ^125^I-induced apoptosis.

### Upregulation of the *JAK/STAT1* Pathway by ^125^I

**Figure 3:**
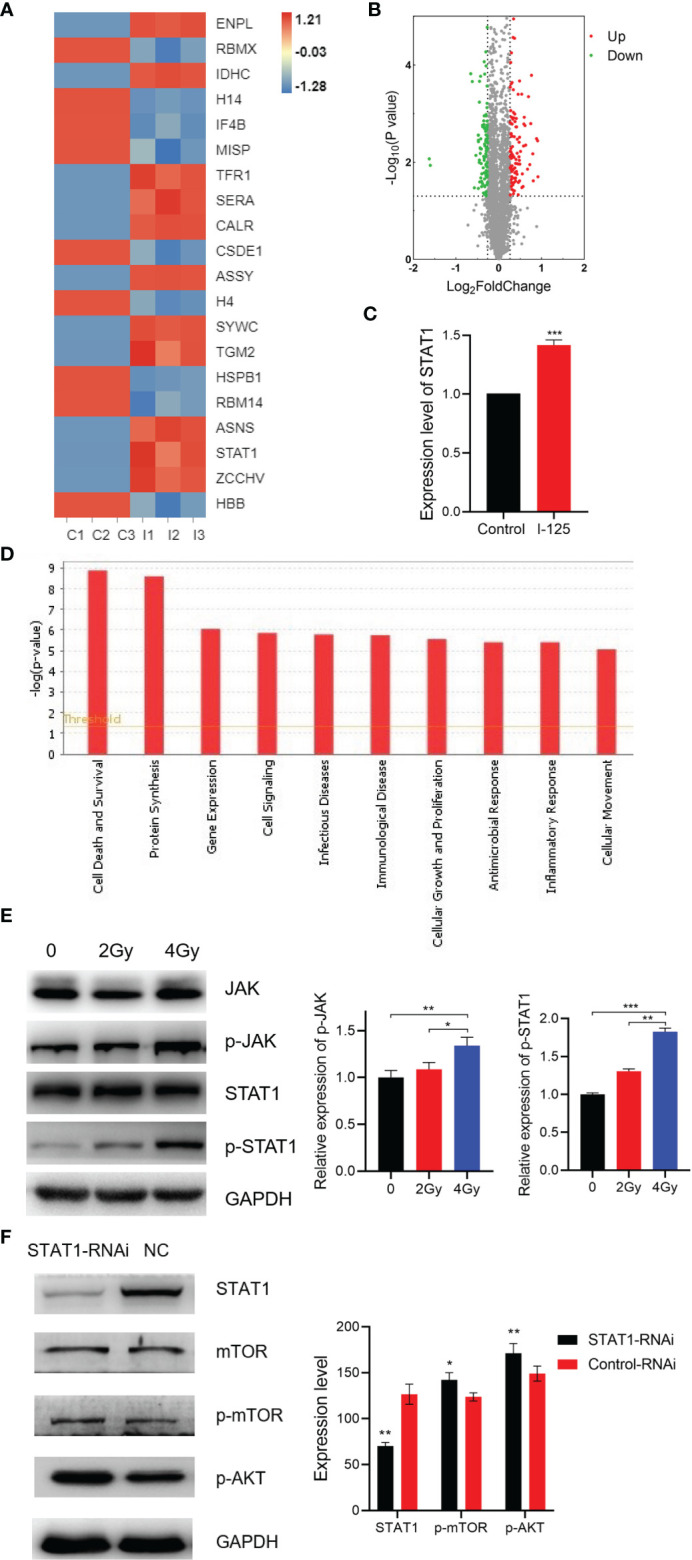
^125^I-induced upregulation of the *JAK/STAT1* pathway. **(A)** Heat map of differentially expressed proteins after treatment with ^125^I in SMMC7721 cells. **(B)** Volcano plot of differentially expressed proteins in SMMC7721 cells. Green represents downregulated proteins and red upregulated proteins. **(C)** The expression level of *STAT1* in iTRAQ assay. **(D)** Analysis results of disease and biofunctions of HCC cells. **(E)** Western blot of the JAK/STAT1 pathway. **(F)** Western blot analysis of AKT/mTOR pathway. All experiments were performed in triplicate, and the data are presented as the mean ± SD. *P < 0.05, **P < 0.01, ***P < 0.001. iTRAQ, isobaric tag for relative and absolute quantification labeling; HCC, hepatocellular carcinoma cells; EPI, epirubicin.

The heat map of iTRAQ labeling, performed using SMMC7721 cells with or without ^125^I treatment to quantify differences in protein expression after ^125^I treatment, is shown in **Figure 3A**. Protein expression levels were significantly different between the ^125^I treatment group and the non-treatment group, with a total of 207 differentially expressed proteins in ^125^I-treated HCC cells, including 119 upregulated and 88 downregulated proteins, as shown in the volcano plot in **Figure 3B**. Of note, the expression level of STAT1 was significantly different between the ^125^I treatment and control groups, as shown by the iTRAQ results (**Figure 3C**). Regarding the disease and function status of HCC cells, ^125^I influenced both cell death and survival, as well as on protein synthesis. These findings are indicative of a specific effect of ^125^I in upregulation of the *JAK/STAT1* pathway, which is involved in regulating the cell state, including survival, and intracellular protein synthesis (**Figure 3D**). Western blot analysis revealed a dose-dependent increase in the expression of *p-JAK* and *p-STAT1* proteins in ^125^I-treated HCC cells (**Figure 3E**). Furthermore, based on our previous study, in order to investigate the potential downstream of *STAT1*, western blot was performed to detect the expression level of *AKT/mTOR* pathway (3). The results showed that *AKT/mTOR* pathway was upregulated when *STAT1* was knock down (**Figure 3F**).

### ^125^I-Induced Apoptosis and Anti-Proliferation of HCC Cells by Means of the *JAK-STAT1* Pathway

**Figure 4:**
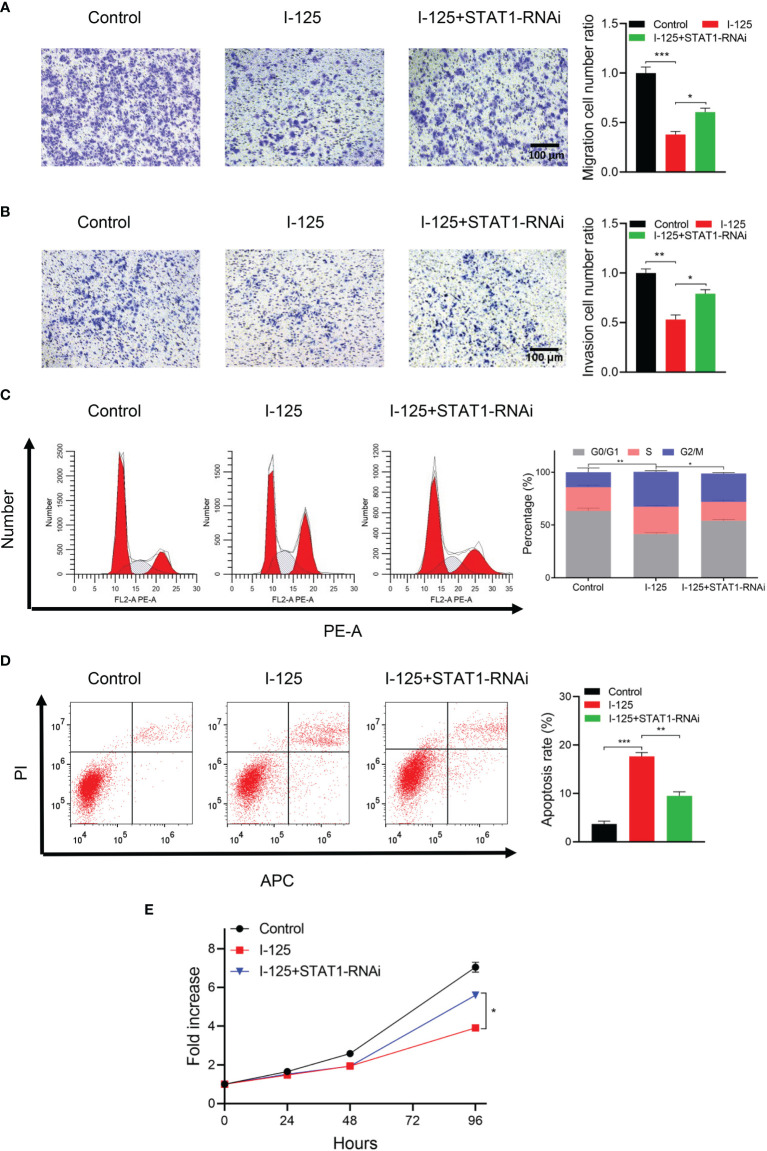
^125^I-induced apoptosis and inhibition of the proliferation of HCC cells *via* the *JAK/STAT1* pathway. **(A)** Transwell assays showing migration and **(B)** invasion of SMMC7721 cells transfected with STAT-RNAi. **(C)** Flow cytometry showing the influence of *STAT1*-RNAi on cell proliferation and **(D)** apoptosis**. (E)** CCK-8 assay showing HCC cell proliferation. All experiments were performed in triplicate, and the data are presented as the mean ± SD. *P < 0.05, **P < 0.01, ***P < 0.001. HCC, hepatocellular carcinoma cells.

In transwell assays of cells transfected with *STAT1*-RNAi or NC-RNAi, there was a significant inhibition of the invasion and migration of SMMC7721 cells after ^125^I irradiation; this ^125^I-induced effect was attenuated by the downregulation of *STAT1* expression (**Figures 4A, B**). The results of flow cytometry, performed to further validate the function of the *JAK-STAT1* pathway in ^125^I-induced anti-proliferation and apoptosis of HCC cells, showed an arrest in the G2/M phase of the cell cycle with ^125^I treatment (**Figures 4C, D**). Thus, ^125^I treatment can inhibit the proliferation of HCC cells, with this effect being attenuated by the downregulation of *STAT1*. It is confirmed by the CCK-8 assay that *STAT1*-RNAi blocked the anti-proliferation effect of ^125^I treatment (**Figure 4E**). Taken together, these results suggest that ^125^I inhibits the proliferation and promotes apoptosis of HCC cells by means of the *JAK-STAT1* pathway.

### Enhancement of the ^125^I-Induced Anti-Cancer Effects by EPI *via* the *JAK/STAT1* Pathway

**Figure 5:**
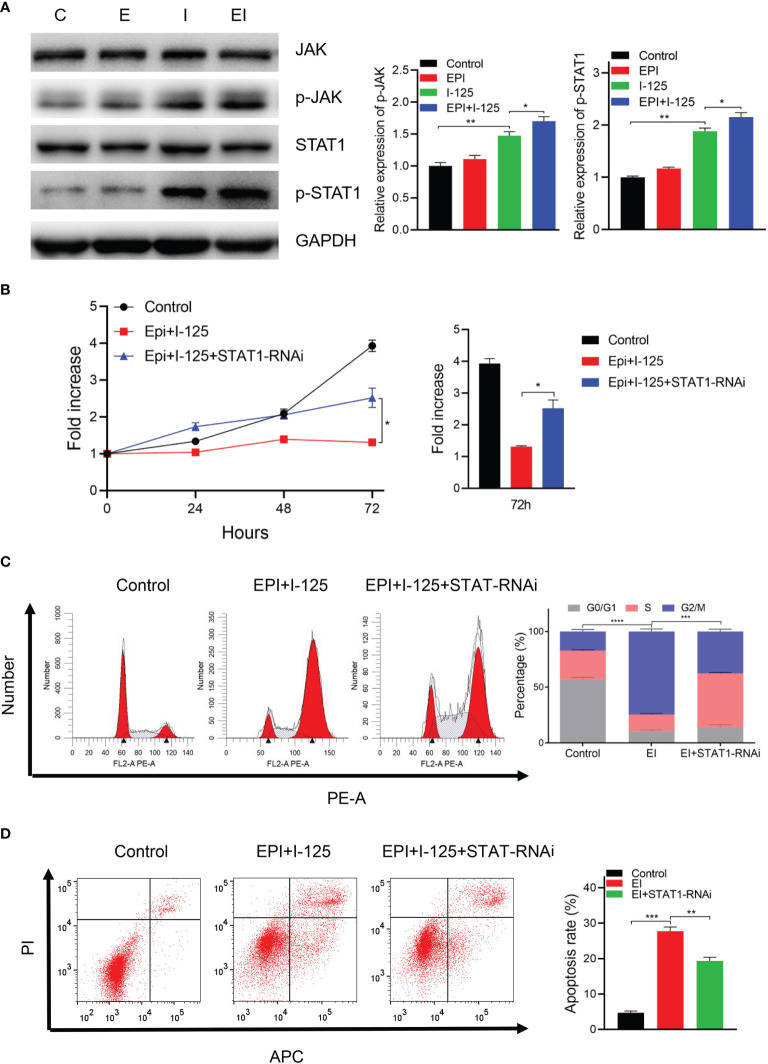
EPI increased the anti-cancer effects of ^125^I *via* the *JAK/STAT1* pathway. **(A)** Western blot showing the expression of *JAK/STAT1* signaling. **(B)** CCK-8 showing cell proliferation ability. **(C, D)** Flow cytometry results showing the effects on cell cycle **(C)** and apoptosis **(D)**. All the experiments were performed in triplicate, and the data are presented as the mean ± SD. *P < 0.05, **P < 0.01, ***P < 0.001, ****P < 0.0001. EPI, epirubicin; CCK-8, cell counting kit-8.

The results of treatment of cells transfected with NC-RNAi or *STAT1*-RNAi with ^125^I or EPI, alone or in combination, performed to evaluate the underlying molecular mechanism by which EPI improved ^125^I-induced proliferation and apoptosis *via* the *JAK-STAT1* pathway, are shown in **Figure 5**. There was no marked change in the total amount of *JAK* and *STAT1* proteins, although there was a greater increase in the phosphorylation status of these two proteins with the combined treatment compared to either single treatment group (**Figure 5A**). Moreover, *in vivo* experiment obtained the same result that the expression level of p-STAT1 was higher in combined treatment group than single group (**Supplementary Figure 1**). To further investigate the possible involvement of the *JAK/STAT1* pathway in the anti-cancer effects of ^125^I and EPI, cell viability was analyzed using CCK-8. The results showed that downregulation of *STAT1* attenuated the anti-cancer effects of ^125^I and EPI (**Figure 5B**). On flow cytometry analysis, downregulation of *STAT1* also attenuated the effect of combined ^125^I and EPI treatment on G2/M cell cycle arrest (**Figure 5C**) and apoptosis (**Figure 5D**). Collectively, these results show that EPI enhances the anti-cancer effect of ^125^I treatment *via* the *JAK/STAT1* pathway.

### Attenuation of ^125^I- and EPI-Induced Anti-Cancer Effects *via* the Downregulation of *STAT1 in vivo*

**Figure 6:**
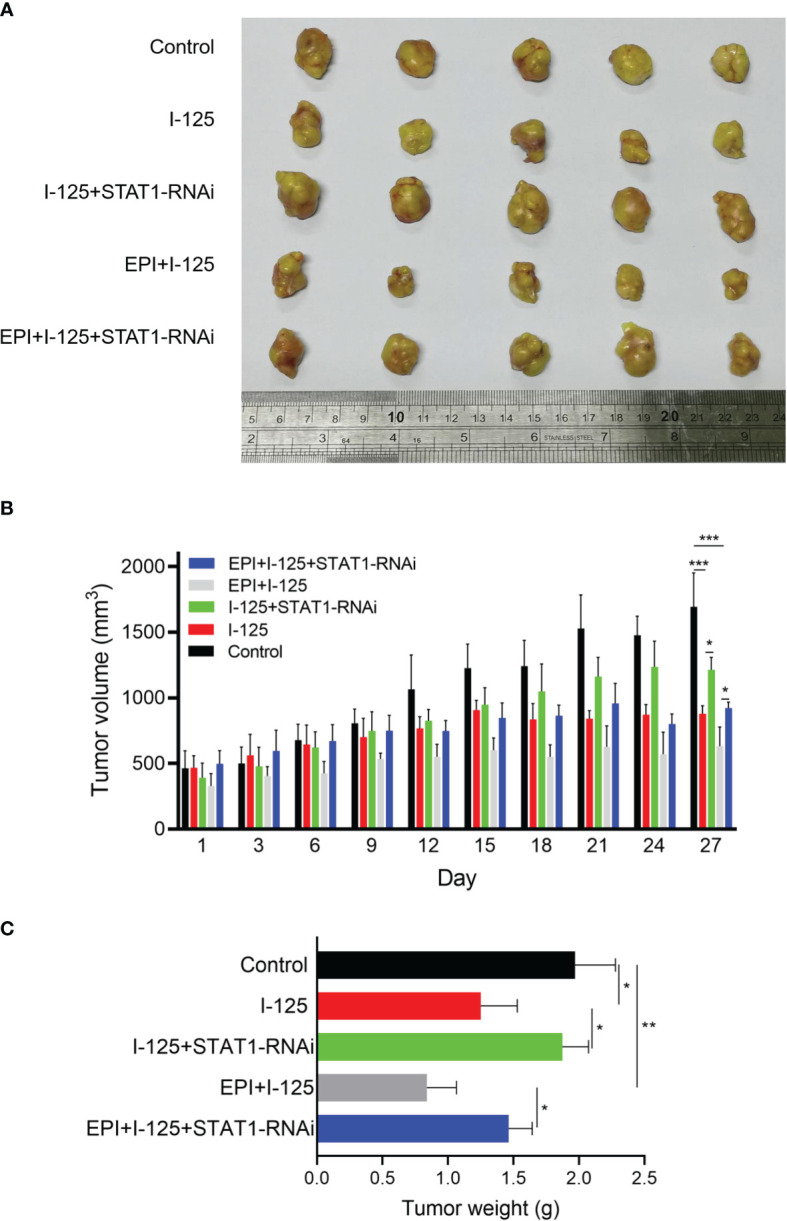
Attenuation of the anti-cancer effect induced by ^125^I and EPI by *STAT1* downregulation *in vivo*. Nude mouse model was created using SMMC7721 cells transfected with NT-RNAi or *STAT1*-RNAi and treated with ^125^I and EPI. **(A)** The tumor models were treated and stripped, showing the volume of 5 tumors in each group. **(B, C)** Tumor volume and weight in each group were measured and the results were shown. The data were expressed as mean ± standard deviation. *P < 0.05, **P < 0.01, ***P < 0.001. EPI, epirubicin.

Treatment of SMMC7721 xenograft tumors with ^125^I, EPI, and *STAT1*-RNAi, performed to further investigate the function of *STAT1* on the ^125^I-induced anti-cancer effect, produced significant suppression of tumor growth by ^125^I (**Figure 6**). This suppression was considerably enhanced by combined ^125^I and EPI, with the combined treatment inducing more obvious anti-cancer effects than either single treatment. The tumor volume in ^125^I was 878.780 ± 61.764 mm^3^, while the combined group of EPI and ^125^I was 630.280 ± 147.418 mm^3^. The inhibition of tumor growth induced by either ^125^I alone or in combination with EPI was compromised when *STAT1*-RNAi was transfected into SMMC7721 cells (**Figure 6A, B**). The tumor volume in *STAT1*-RNAi combined with ^125^I alone or in combination with EPI was 1212.240 ± 96.013 and 921.160 ± 45.790 mm^3^. Therefore, the knockdown of *STAT1* weakened the anti-cancer effect of ^125^I. The effects of ^125^I, EPI, and *STAT1*-RNAi on tumor growth were confirmed by tumor weight measurements (**Figure 6C**). Therefore, *STAT1* downregulation attenuated the anti-cancer effects induced by ^125^I and EPI *in vivo*.

## Discussion

The findings of our study confirm our hypothesis that EPI can promote the anti-cancer effects of ^125^I seed implantation on HCC cells *via* the *JAK/STAT1* pathway by enhancement of HCC cell apoptosis, inhibition of cell proliferation and migration, and arrest of the cell cycle at the G2/M phase.

Previous studies have reported the curative effect of ^125^I radioactive seed implantation for HCC (14–16). Zhang et al. (17) demonstrated that ^125^I radioactive seed implantation improved the expression of main histocompatibility complex class I chain-related gene A in HCC cells and upregulated cytokine-induced killer cell-mediated apoptosis *via* activation of caspase-3. There is also accumulating evidence that ^125^I seed implantation inhibits metastasis and tumor growth by regulating miRNAs. The use of lobaplatin can promote the radiosensitivity of HCC and NSCLC to ^125^I seeds (3, 4). Additionally, ^125^I can upregulate the expression of the *PERK-eIF2α-ATF4-CHOP* pathway to inhibit proliferation and accelerate apoptosis of HCC cells (3, 18). Of note, ^125^I can promote the downregulation of *p38MAPK* and the degradation of *MDM2* in NSCLC, thereby inducing apoptosis (19). Thus, ^125^I seed implantation inhibits tumor invasion by changing the expression levels of vimentin, N-cadherin, and *MMP-9* and induces the apoptosis of NSCLC cells *via* its effect on the mitochondrial pathway (20).

In a previous study on pancreatic cancer, we reported that ^125^I seed implantation combined with gemcitabine improved the ratio of *Bax/Bcl-2* in pancreatic cancer cells, yielding a clinically better anti-proliferation effect (15). A recent study confirmed the above conclusion that *ING4* gene therapy combined with ^125^I seed implantation effectively inhibited the growth and angiogenesis of pancreatic cancer (21). Similarly, prostate brachytherapy using ^125^I seeds effectively prolonged life and significantly improved the quality of life of patients with prostate cancer (22). In summary, ^125^I seed implantation has significant efficacy for the treatment of various cancers; thus, further studies to clarify the mechanism by which ^125^I seed implantation inhibits malignant proliferation of tumor cells will be key to better defining the use of ^125^I seed implantation for tumor treatment.

EPI is an anthracycline drug that has been widely used in the clinical treatment of NSCLC, breast, liver, and stomach cancer (10, 11, 13, 23). EPI inhibits the proliferation of cells by embedding itself directly in the cell DNA, thus interfering with the transcription process, inhibiting mRNA synthesis and topoisomerase II activity, and producing oxygen and free radicals (10, 12). As a traditional chemotherapy drug, EPI has a significant effect in the adjuvant treatment of early breast cancer, mainly by inhibiting the metastasis of breast cancer cells, thus improving the prognosis of patients with breast cancer (10, 24). EPI is less cardiotoxic than other adriamycin drugs, and when combined with trastuzumab, paclitaxel, and other chemotherapy drugs, it can significantly improve the clinical efficacy of tumor treatment (12, 13). EPI can also be loaded on other carriers such as polymeric micelles and hyaluronic acid to inhibit tumor proliferation (12, 13). In clinical applications, TACE combined with EPI has a significant effect on improving prognosis and prolonging the survival of patients with HCC (25). Based on this evidence, we explored the sensitization effect of EPI on ^125^I seeds both *in vivo* and *in vitro*. Our findings are in line with those of a previous study (12), proving that EPI inhibits the proliferation of HCC cells and promotes ^125^I-induced apoptosis.

*STAT1* is a member of the *STAT* family and participates in signal transduction inside and outside cells and the regulation of gene transcription in the nucleus (6). As any abnormality or change in signal regulatory factors can lead to tumor formation, the role of *STAT1* in the occurrence and development of tumors requires further study. There is evidence that *STAT1* may play a dual role in this regard (8, 26). Specifically, while there is some evidence that *STAT1* can induce tumorigenesis, accumulating evidence has shown that *STAT1* is a tumor suppressor, exerting its anti-tumor role by interfering with the tumor microenvironment and/or signaling pathway (7, 26, 27). Previous studies have shown that *STAT1* can participate in antiviral and immune defense and promote cell apoptosis and inhibit tumor growth by regulating anti-apoptotic genes such as *BCL-xL*, caspases, and *Bax* (7, 8). Consistent with our findings that increased proliferation, invasion, and migration of the HCC cell line SMMC-7721 were significantly inhibited after transfection of *STAT1*-siRNA into HCC cells, inhibition of *STAT1* expression was reported to promote metastasis of osteosarcoma (27). *STAT1* was also found to mediate an important anti-tumor response for squamous cell carcinoma of the head and neck (28), with increased *STAT1* expression inhibiting the progression of ovarian cancer (29) and improving the prognosis of both of these cancers. *STAT1* has been confirmed to be under-expressed in tissue specimens of HCC, ovarian, and lung cancer and other solid tumors (7, 30). In this study, we used a schematic illustration to show the important role of STAT1 in regulating the apoptosis, proliferation, and metastasis effect in the combined treatment of ^125^I and EPI in HCC, which indicating the value of *STAT1* as a latent biomarker and prognostic indicator for HCC (**Supplementary Figure 2**).

The limitations of our study should be acknowledged in the interpretation of our results. First, more HCC cell lines should be used to verify the results. Second, as *STAT1* is a transcription factor, its potential targets and detailed functions should be investigated in future studies.

In conclusion, this study provided evidence for the role of EPI in promoting ^125^I-induced anti-cancer effects in HCC. Furthermore, the effects of ^125^I and EPI are mediated by the *JAK/STAT1* pathway. As such, the *JAK/STAT1* pathway is a potential target for ^125^I seed implantation in the treatment of HCC.

## Data Availability Statement

The original contributions presented in the study are included in the article/**Supplementary Material**. Further inquiries can be directed to the corresponding authors.

## Ethics Statement

The animal study was reviewed and approved by Shandong University.

## Author Contributions

LG: methodology, project administration, writing of original draft; JS and CW: supervision, investigation, data curation; YaW and YgW: resources and investigation; DL: supervision, project administration, investigation, writing review and editing; YL: funding acquisition, writing review, and editing, supervision, project administration, investigation.

## Funding

This study is supported by Natural Science Foundation of Shandong Province (ZR202102230729), National Natural Science Foundation of China (12171285&11971269), Fund from Shandong Provincial Department of Finance (S190009280000), Key Research & Development Plan of Shandong Province (2019GSF108105), Science and technology project of (Sinopec Group) Shengli Petroleum Administration Co., Ltd (GKY2001), Science and technology project of Jinan Health Committee (2020–3–65), Traditional Chinese medicine scientific research project of Weifang Health Commission (2021-2-002), and Beijing Medical Award Foundation (YXJL-2021-0353-0625).

## Conflict of Interest

The authors declare that the research was conducted in the absence of any commercial or financial relationships that could be construed as a potential conflict of interest.

## Publisher’s Note

All claims expressed in this article are solely those of the authors and do not necessarily represent those of their affiliated organizations, or those of the publisher, the editors and the reviewers. Any product that may be evaluated in this article, or claim that may be made by its manufacturer, is not guaranteed or endorsed by the publisher.
