# Supplementary figures and images for: Epirubicin Enhances the Anti-Cancer Effects of Radioactive 125I Seeds in Hepatocellular Carcinoma via Downregulation of the JAK/STAT1 Pathway
Source: Front Oncol. 2022 May 27;12:854023. doi: 10.3389/fonc.2022.854023 (PMC9184686; doi:10.3389/fonc.2022.854023)

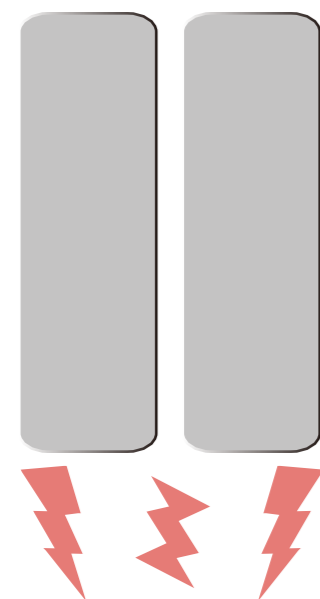

I-125

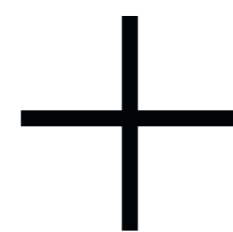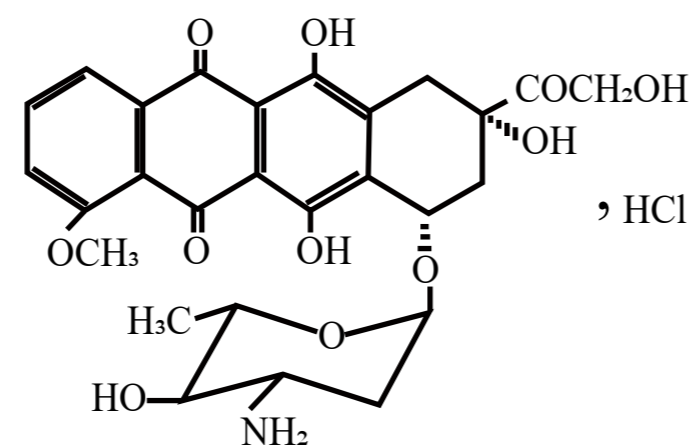

EPI

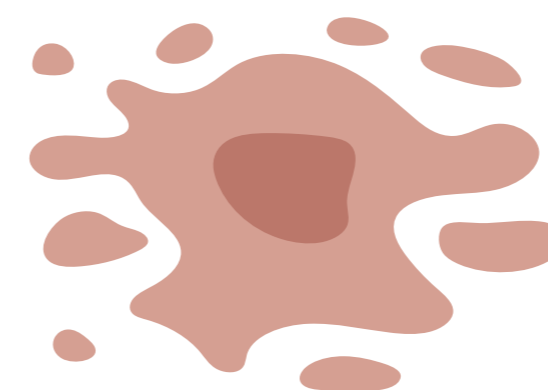

Apoptosis

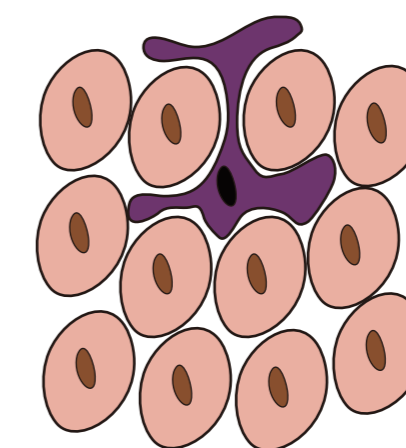

Metastasis

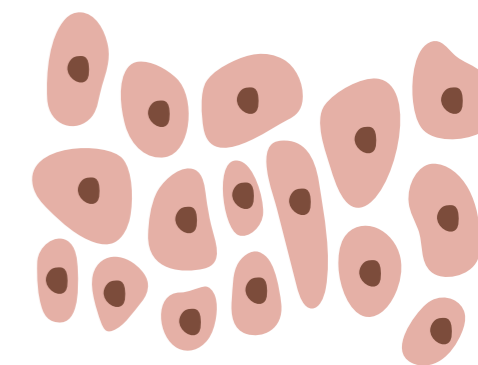

Proliferation

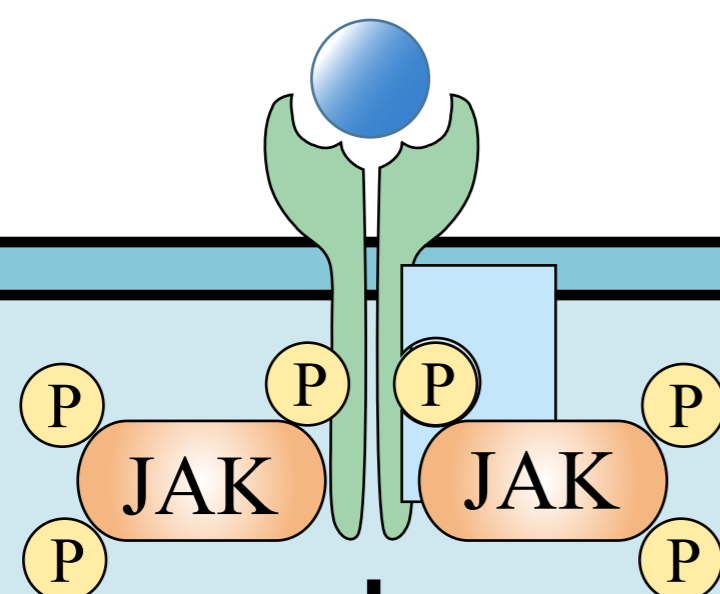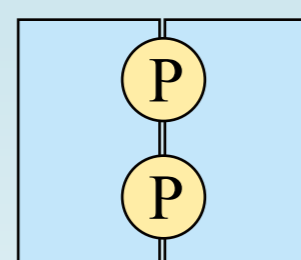

STAT1

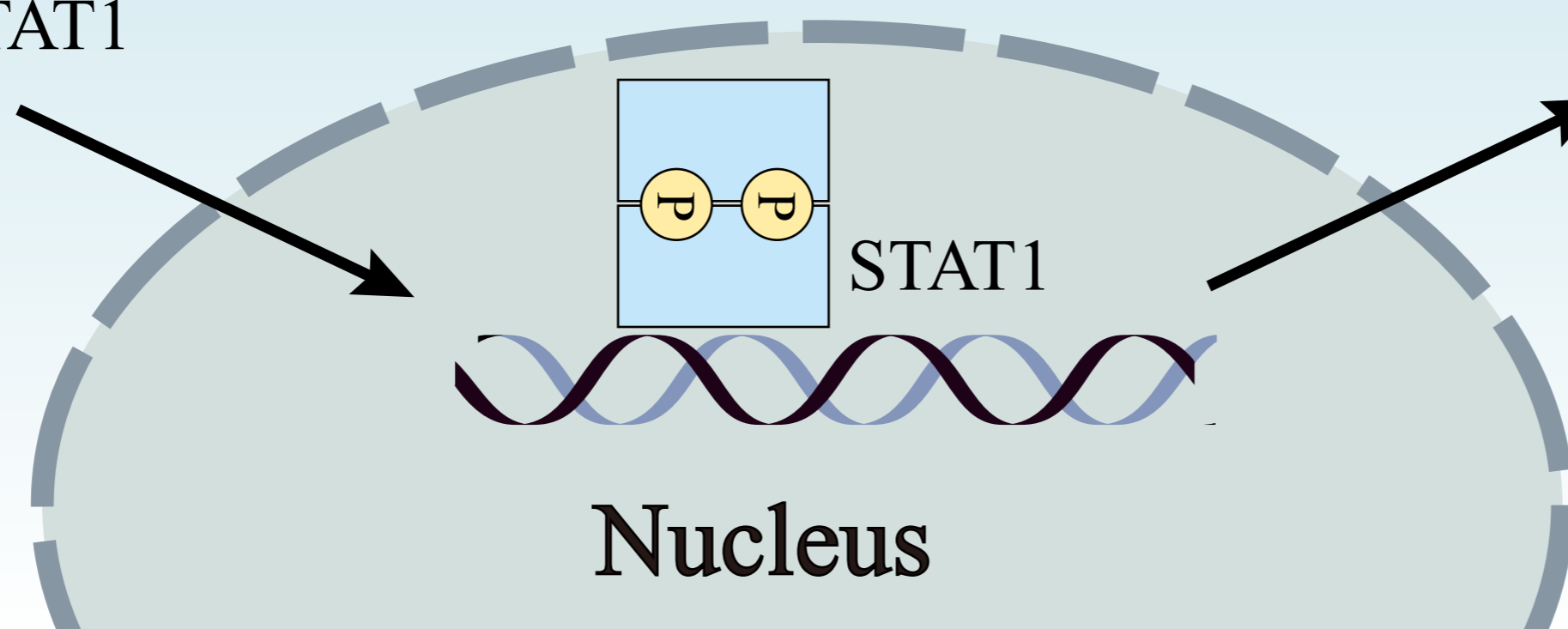

Nucleus

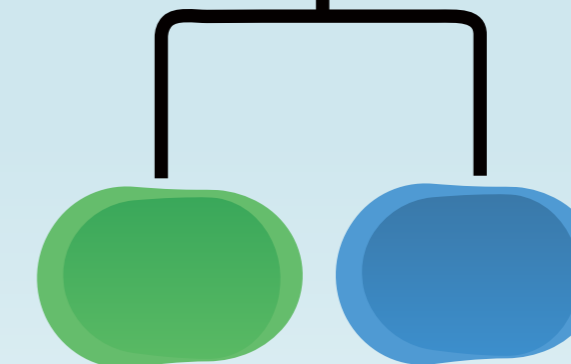

Bax ↑ / Bcl2 ↓

Cytoplasm

Supplement: Supplementary file 1 [file Image_1.pdf]

p-STAT1

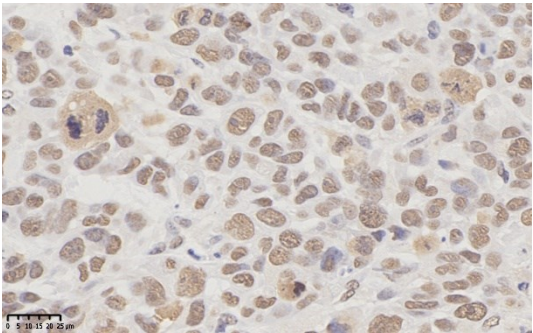

C

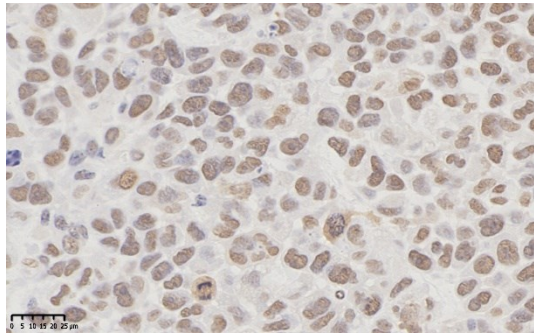

E

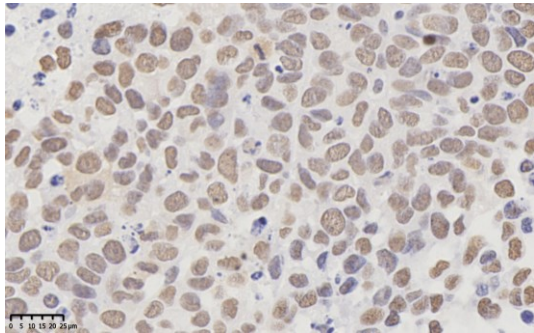

I

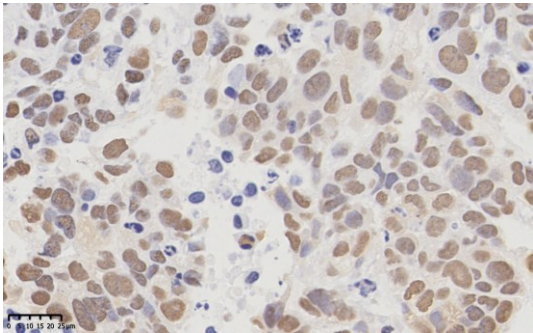

EI

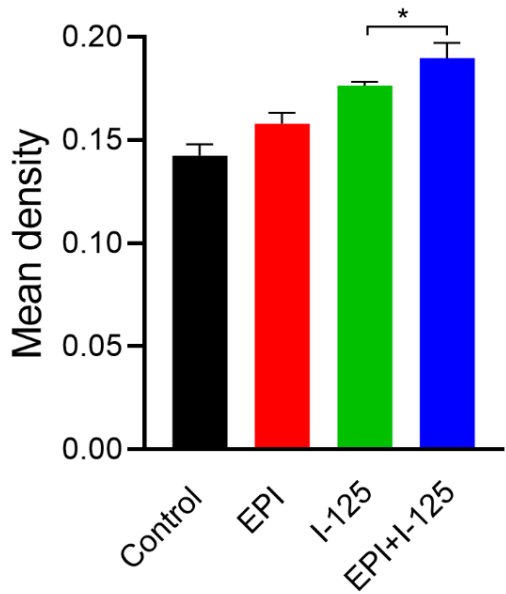

Supplement: Supplementary file 2 [file Image_2.pdf]
